# Supplementary material for: The genome sequence of the Frosted Green moth, Polyploca ridens (Fabricius, 1787)
Source: Wellcome Open Res. 2024 Sep 24;9:545. [Version 1] doi: 10.12688/wellcomeopenres.23043.1 (PMC13213368; doi:10.12688/wellcomeopenres.23043.1)
Supplement: Peer review history

# Reviewer report: Reviewer response for version 1

**DOI:** 10.21956/wellcomeopenres.25376.r131797

**Published:** 2026-05-26

**Authors:** Gulab D Khedkar

**Affiliations:** 1 Dr. Babasaheb Ambedkar Marathwada University, Aurangabad, Maharashtra, India

The manuscript, “The genome sequence of the Frosted Green moth, Polyploca ridens (Fabricius, 1787)” presents a high-quality chromosomal-level genome assembly of the Frosted Green moth, *Polyploca ridens*. The study provides an important genomic resource for Lepidopteran research, particularly for the comparatively understudied family Drepanidae. The manuscript is clearly written, technically sound, and methodologically rigorous. The authors successfully assembled a genome of approximately 493.6 Mb, with most of the assembly scaffolded into 30 chromosomal pseudomolecules, including the Z chromosome. The assembly quality metrics are impressive, including a scaffold N50 of 16.8 Mb, BUSCO completeness of 95.1%, and k-mer completeness of 100%, demonstrating a highly complete and reliable assembly. The integration of PacBio HiFi long reads with Hi-C scaffolding and comprehensive annotation strategies further strengthens the robustness of the dataset. One of the major strengths of this manuscript is the transparency and reproducibility of the workflow. The authors provide detailed methodological descriptions, software versions, accession numbers, and publicly accessible datasets, ensuring that the study can serve as a valuable reference for future genomic and evolutionary studies. The inclusion of DNA barcoding-based specimen verification also adds confidence to the taxonomic accuracy of the assembly.

 The background section effectively highlights the ecological significance and conservation relevance of *P. ridens*, especially considering habitat-associated declines in parts of Europe. Although the manuscript is primarily a genome note and not intended to explore extensive functional genomics, the availability of this resource will undoubtedly facilitate future studies on Lepidopteran evolution, adaptation, comparative genomics, and conservation biology.

 I have only a few minor suggestions for improvement:

1. The discussion could briefly elaborate on how this genome resource may contribute specifically to comparative genomic analyses within Drepanidae or other Lepidopteran lineages.
2. A short statement regarding possible future applications of the annotated gene set (e.g., adaptation, host-plant interactions, or phylogenomics) would further enhance the broader impact of the manuscript.
3. Some figure legends, particularly those related to assembly visualization tools, may benefit from minor simplification for readers less familiar with genome assembly metrics.

These suggestions are minor and do not affect the scientific quality or integrity of the work.

 Overall, this manuscript represents a valuable contribution to insect genomics and biodiversity research. The dataset is comprehensive, technically robust, and openly accessible. I recommend this manuscript for acceptance after minor editorial consideration.

Are sufficient details of methods and materials provided to allow replication by others?

Yes

Is the rationale for creating the dataset(s) clearly described?

Yes

Are the datasets clearly presented in a useable and accessible format?

Yes

Are the protocols appropriate and is the work technically sound?

Yes

Reviewer Expertise:

Genomics, genome assembly, genome size evaluation, etc

I confirm that I have read this submission and believe that I have an appropriate level of expertise to confirm that it is of an acceptable scientific standard.

# Reviewer report: Reviewer response for version 1

**DOI:** 10.21956/wellcomeopenres.25376.r106443

**Published:** 2024-10-29

**Authors:** Jose Martinez

**Affiliations:** 1 Division of Plant Industry/Entomology, Florida Department of Agriculture and Consumer Services (Ringgold ID: 70124), Gainesville, Florida, USA

Boyes and Crowley have developed a highly complete and well-assembled genome for the enigmatic Frosted Green moth ( *Polyploca ridens*; Fabricius, 1787), underpinned by a clear and well-structured rationale. Their approach is characterized by concise, comprehensible descriptions and meticulously selected protocols that ensure precision and reproducibility. This standardized, richly illustrated dataset is readily accessible, providing an invaluable resource for a wide range of research applications and advancing our understanding of this drepanoid species.

 This exceptionally complete and well-assembled genome, derived from sequencing a male specimen, is organized into 30 chromosomal pseudomolecules (comprising 29 autosomes and the Z sex chromosome). With an outstanding BUSCO score, this meticulously constructed assembly provides a highly detailed and reliable genomic resource.

 As with other genome descriptions, the authors emphasize the conservation importance of this species due to habitat loss. However, it is especially noteworthy that this group of moths, Drepanoidea, remains one of the least studied within Lepidoptera. Consequently, this genomic resource holds value not only for conservation efforts but also as a foundation for broader evolutionary and ecological studies.

Are sufficient details of methods and materials provided to allow replication by others?

Yes

Is the rationale for creating the dataset(s) clearly described?

Yes

Are the datasets clearly presented in a useable and accessible format?

Yes

Are the protocols appropriate and is the work technically sound?

Yes

Reviewer Expertise:

Evolutionary Biology, Ecology, IPM, Integrative Biology, Entomology, Genomics

I confirm that I have read this submission and believe that I have an appropriate level of expertise to confirm that it is of an acceptable scientific standard.
